# *Aegle marmelos* (L.) Correa: An Underutilized Fruit with High Nutraceutical Values: A Review

**DOI:** 10.3390/ijms231810889

**Published:** 2022-09-17

**Authors:** Niharika Sharma, Radha, Manoj Kumar, Baohong Zhang, Neeraj Kumari, Daljeet Singh, Deepak Chandran, Tanmay Sarkar, Sangram Dhumal, Vijay Sheri, Abhijit Dey, Sureshkumar Rajalingam, Sabareeshwari Viswanathan, Pran Mohankumar, Marthandan Vishvanathan, Sangeetha Kizhakkumkara Sathyaseelan, Jose M. Lorenzo

**Affiliations:** 1 School of Biological and Environmental Sciences, Shoolini University of Biotechnology and Management Sciences, Solan 173229, India; 2 Chemical and Biochemical Processing Division, ICAR–Central Institute for Research on Cotton Technology, Mumbai 400019, India; 3 Department of Biology, East Carolina University, Greenville, NC 27858, USA; 4 Department of Veterinary Sciences and Animal Husbandry, Amrita School of Agricultural Sciences, Amrita Vishwa Vidyapeetham University, Coimbatore 642109, India; 5 Department of Food Processing Technology, Malda Polytechnic, West Bengal State Council of Technical Education, Government of West Bengal, Malda 732102, India; 6 Division of Horticulture, RCSM College of Agriculture, Kolhapur 416004, India; 7 Department of Life Sciences, Presidency University, 86/1 College Street, Kolkata 700073, India; 8 Department of Agronomy, Amrita School of Agricultural Sciences, Amrita Vishwa Vidyapeetham University, Coimbatore 642109, India; 9 Department of Soil Science and Agricultural Chemistry, Amrita School of Agricultural Sciences, Amrita Vishwa Vidyapeetham University, Coimbatore 642109, India; 10 School of Agriculture and Biosciences, Karunya Institute of Technology and Sciences, Coimbatore 641114, India; 11 Department of Seed Science and Technology, Amrita School of Agricultural Sciences, Amrita Vishwa Vidyapeetham University, Coimbatore 642109, India; 12 Department of Plantation Crops and Spices, Kerala Agricultural University, Vellanikkara, Thrissur 680656, India; 13 Centro Tecnológico de la Carne de Galicia, Rúa Galicia N° 4, Parque Tecnológico de Galicia, San Cibrao das Viñas, 32900 Ourense, Spain; 14 Área de Tecnología de los Alimentos, Facultad de Ciencias de Ourense, Universidad de Vigo, 32004 Ourense, Spain

**Keywords:** *Aegle marmelos* (Bael), nutrition, phytochemical, pharmacological properties, industrial applications

## Abstract

*Aegle marmelos* (L.) Correa (Bael) fruit, a member of the Rutaceae family, is a major cultivated fruit plant in tropical and subtropical regions in countries of southeast Asia. Bael fruit has been a major topic for studies in recent years mainly due to its high nutritional (carbohydrates, proteins, minerals, and vitamins) value and presence of various phytochemicals, which attributed to its high medicinal value. These phytochemicals include various compounds, e.g., alkaloids, flavonoids, and phenolic acids (protocatechuic acid, gallic, and ellagic acid). The fruit extract of bael has been also an important study area for its pharmacological activities, including antidiarrheal, antioxidant, antidiabetic, hepatoprotective, radioprotective, anticancer, antiulcer properties. The current review mainly highlighted the nutritional and pharmacological activities of bael fruit. The nutritional profile and phytochemical profile were discussed in the review, along with their concentration in the fruit. Moreover, the experiments carried out in vivo and in vitro of bael fruit extracts with respect to their pharmacological activities were also discussed in the article. The recent literature based on nutritional and pharmacological values of bael fruit showed its high potential as a food and pharmaceutical product. Despite having high nutritional and pharmacological value, research related to molecular mechanisms of bael fruit is still limited, and clinical trials are needed to ensure its safety as a product in the food and pharma industries.

## 1. Introduction

**Figure 1:**
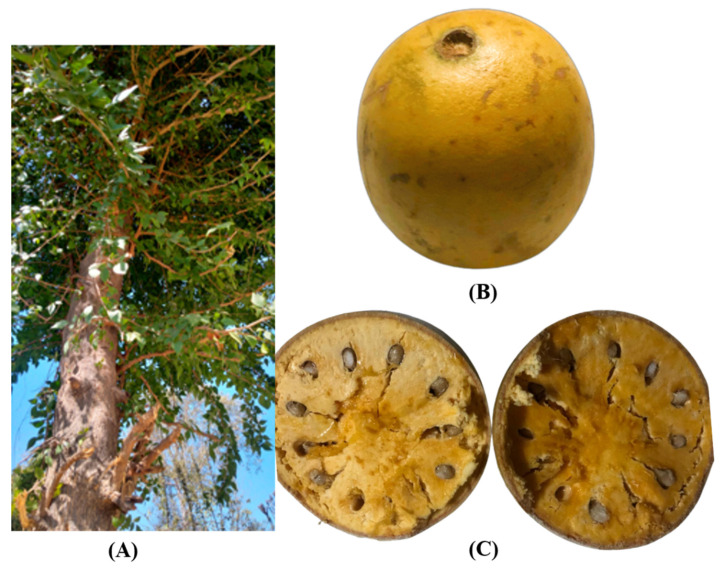
*Aegle marmelos* (L.) Correa tree (**A**) with whole fruit (**B**) and (**C**) cross-cut fruit.

*Aegle marmelos* (L.) Correa (Bael), important member of plant family Rutaceae, native to Eastern Ghats and Central India, is widely utilized in traditional medicines due to its excellent therapeutic characteristics. Although being a subtropical tree it can grow well in tropical climates too at an altitude of 1200 m. Bael is widely distributed throughout Indian Peninsula and grows in most of the southeast Asian countries [1,2,3]. The bael fruits exhibit a diverse range of shape from round, oval, pyriform, or oblong and are 5–25 cm in diameter, also the seeds can be from 10 to 50 in number having flat-oblong shape and 1 cm long [4] (Figure 1). Although having high moisture content of nearly 61%, bael fruits have high nutritional composition as it contains minerals (phosphorus, potassium, calcium, magnesium, iron, copper, zinc, chromium), fat, fiber (hemicellulose, cellulose, lignin, pectin), protein, carbohydrate, vitamins (B_1_, B_2_, B_3_, C), amino acids (threonine, valine, methionine, isoleucine, leucine, lysine), and fatty acids [5,6,7,8]. Phytochemical profiling of bael fruit showed that it also contains many useful bioactive compounds and phytochemicals, which include polyphenols, coumarins (alloimperatorin, zanthotoxol, imperatorin, xanthotoxol, isoimperatorin, umbelliferone, marmelide, scopoletin, marmelosin, scopolentin, marmesin, psoralen-a, scoparone, marmin, methyl ether, psoralen); tannins (4,7,8-trimethoxyfuro-quinoline, skimminianine); alkaloids (aegelenine, halfordinol, aegeline, ethyl cinnamate, aegelinosides A, ethyl cinnamamide, aegelinosides B, dictamine, fragrine); phenolic acids (gallic acids, p-coumaric acid, 2,3-dihydroxy benzoic acid, vanillic acid, chlorogenic acid); organic acids; flavonoids (rutin); tocopherols; and carotenes [9,10,11,12]. Being as strong antioxidants, the phytochemical and nutritional components are involved in a variety of biological processes, making it a potential food ingredient [13]. Various studies have shown many bioactivities of bael fruit, which includes antidiarrheal, antioxidant, antidiabetic, hepatoprotective, radio protective, anticancer, and antiulcer activities showing a high potential in pharma products [14]. The bael fruits in ripe form is considered as astringent, tonic, laxative, restorative, and brain and heart. The unriped fruits of *A. marmelos* are in the treatment of dysentery and also diarrhea diarrhea and dysentery as it is an astringent, digestive and stomachic [15].

Bael fruits have many uses in functional foods also as it has much potential for processing into goods such as preserves, powder, jam, wine, slab, and syrup [1]. In India many of the products are prepared from bael fruits such as bael sherbet, murabba or syrups. In other countries such as Indonesia and Thailand ripe bael fruits and their sliced pieces are consumed as food and syrups are used in making cake ingredients [16]. The processing of bael fruit also produce many waste materials such as seeds, fibers, peel, etc. which also contain many bioactive and pharmaceutical compounds [17,18]. From various regions of India, The Indian Council of Agricultural Research (ICAR) and Agricultural institutes are developing promising varieties of bael through selection in the recent years [8]. The nutritional and phytochemical profile of bael fruits is highlighted in this review with the goal of increasing its use as a food ingredient. Furthermore, the biological activity of bael fruit will be thoroughly discussed including its use as a functional ingredient in foods.

## 2. Nutritional Composition

### 2.1. Polysaccharides

Polysaccharides plays a very important role in contribution to the nutritional value of fruits and are also the major component of human diet [19,20]. In a study, nutritional constituents of bael were investigated from the fruit pulp at different development stages of premature fruits to mature. The carbohydrate and total sugar content were estimated to be 36.80–41.70% and 3.08–6.94%, respectively. The carbohydrate content was found to be highest in premature stages of fruits whereas total sugar content being highest at later mature stages [21]. In a similar study of estimation of important constituents of bael fruit during various ripening stages, the fructose, glucose, and sucrose content in bael fruit was found to be in range of 1.01–1.55%, 1.15–1.88%, and 2.45–12.01%, respectively [22]. In another study of physicochemical analysis of bael fruit, the total sugar content was estimated in fruit pulp and results showed 14.35% of total sugars, majorly contributed by non-reducing sugars, i.e., 9.93%, which is more than double the content of reducing sugars, which was found to be 4.42% (Table 1). In comparison to fruit pulp, pericarp shows less sugar content of 1.83%, contributed evenly by both reducing (0.92%) and non-reducing sugars (0.91%) [6]. In a similar study of nutritional exploration of bael fruit, total carbohydrate content was estimated to be 34.35% in fruit pulp [5]. In a study proximate composition of bael fruit pulp was estimated and results showed total soluble sugars content of 7.6 g/100 g, mainly contributed by reducing sugars, i.e., 6.2 g/100 g and less contributed by non-reducing sugars, i.e., 1.4 g/100 g. The starch content was also estimated and found to be 3.6 g/100 g in bael fruit pulp [23]. In an independent study of nutrient content estimation of bael fruit, the results showed that bael fruit contain 31.8% of carbohydrate content [24]. The various studies showed that bael fruit has high nutritional value and is a significant source of polysaccharides.

### 2.2. Proteins

The nutritional value is determined by the protein quality and quantity, and fruits have low protein content [32,33]. In a recent study of nutritional evaluation of bael, the fruit was investigated for protein content, and results showed 7.52–8.81% protein in fruit pulp at different stages of development. The highest protein content was at the premature stage, i.e., 8.81%, while the lowest was at the mature stage, i.e., 7.52% [21]. In another study, physicochemical analysis of bael was analyzed, and protein content was estimated to be 3.64% and 1.31% in fruit pulp and pericarp, respectively [6]. Sharma and Chauhan [25] evaluated bael fruit for its nutritional value, and interestingly, the results showed 4.35% of the protein in powdered bael fruit pulp. In a similar study of nutritional evaluation of fruits of bael, the crude protein content was estimated to be 1.87% in fruit pulp [5]. The analysis study of physicochemical composition carried out on fresh bael fruits of two different cultivars yielded differences in protein yield results, which were 3.6% for the local cultivar and 8.8% for the NB-5 cultivar of bael fruit [1]. In another study of proximate composition analysis of bael, the results showed crude protein content of 4.7 g/100 g in bael fruit pulp [23]. In a study related to the nutritional analysis of bael, the protein content was estimated to be 1.8% in bael fruit [24]. Similarly, another study by Kumar and co-workers [26] also reported the same average protein value upon the analysis of bael fruits.

### 2.3. Essential Oils/Lipid Profile

Lipids are generally described as fats and oils. At room temperature, fats are solid, whereas oil is in liquid form. Lipids such as fats, oils, fatty acids, and greases are the essential components of natural foods and many synthetic compounds [34]. A study discovered that the pulp of the bael fruit contains a number of restricted amino acids, with lysine having the highest amino acid score of 47, followed by valine (52), threonine (53), and isoleucine (97). Methionine and leucine, on the other hand, were numerous, with amino acid scores of 255 and 317, respectively. Saturated fatty acids made up 60.67% of fatty acids in bael plant seeds, but only 45.61 and 18.31% in fruit pulp and leaves, respectively. Palmitoleic acid (C16:0) was discovered to be the most abundant fatty acid in all portions of the bael plant [5]. Different studies indicated that the edible portion of *A. marmelos* fruit comprises 0.6% fat and that the fruit includes a significant number of proteins and little fat. The nutritional composition of powder was found to have a substantial (*p* < 0.001) concentrated effect on edible fruit composition. According to Gopalan [28], the bael fruit pulp has 3.7% fat, while the current analysis showed a fat level of 3.2% [27,28]. Essential oils are volatile and aromatic liquids extracted from plant material. Under normal conditions, these aromatic compounds are chemically pure and volatile [35,36]. According to a study, the yield and content of essential oils vary seasonally [37]. A recent study depicted that *A. marmelos* seeds contain 34.4% oil, which is utilized in aromatherapy, cosmetics, and compressors. Anti-inflammatory, antibacterial, antiseptic, antioxidant, carminative, astringent, cytophylactic, and disinfectant properties can all be found in *A. marmelos* oil [38].

### 2.4. Minerals and Vitamins

Bael fruit is a rich source of a variety of nutrients that are useful for human health since it includes a number of vitamins and minerals. Because it is abundant in vitamins, including vitamin A, vitamin B complex, and vitamin C, bael has been discovered to work as an antioxidant, thus preventing rancidity and color loss [39,40]. The minerals reported from the part of bael include calcium, iron, phosphorus, potassium, and salts. Kumar et al. [26] reported that unripe fruit is more beneficial for medicinal purposes than ripe fruit. It includes mineral (1.9%), potassium (610 mg), phosphorus (52 mg), calcium (80 mg), fiber (2.9%), carotene (55 mg) and protein (1.6%), in fruit juice [1,7]. In another study, it was found that bael fruit nutrients are extremely beneficial for human health, and this is already proved by various researchers by conducting various investigations on bael fruit. The main constitution of *A. marmelos* nutrients is fatty acids, vitamins, glucose, amino acids, and minerals [39]. It can prevent color loss and rancidity because it contains a valuable amount of vitamin A (55 mg), vitamin C (8 mg), and vitamin B, which can act as a potential antioxidant agent [38]. Fruit pulp of *A. marmelos* comprises of calcium (80 mg), mineral content (1.7%), phosphorous (52 mg), copper (0.21 mg), potassium (610 mg), and iron (0.60 mg/100 g) [1]. The calorific value of bael fruit (88 cal/100 g) is higher than that of mango (36 cal/100 g), apple (64 cal/100 g), and guava (59 cal/100 g) [38,40]. In a separate study, it was found that it is also high in vitamins such as riboflavin (1190–1200 mg/100 g), vitamin B_1_ (0.13 mg), vitamin A (55 mg), vitamin B_2_ (1200 mg), ascorbic acid (8 mg/100 g), vitamin C (8 mg) and thiamine (0.13 mg) [30,31]. In another study, bael fruit pulp was reported for numerous vitamin concentrations, including vitamin B_1_ (0.16 mg%), vitamin C (73.2 mg%), vitamin B_2_ (0.18 mg%), and vitamin B_3_ (0.87 mg%). According to vitamin analysis, the bael is recognized as a suitable source of ascorbic acid and several vitamins of the B group. Vitamin C concentration was found to be 73.2 mg/100 g, which was significantly higher than that found in Thai bael fruit (26.17 mg/100 g) and bael fruit growing under Indian conditions (40 mg/100 g). Vitamin C levels in unripe bael fruit are relatively high (620 mg/100 g) [5]. Furthermore, vitamin C (8–60 mg), riboflavin (1.19 mg), vitamin A (55 mg), thiamine (0.13 mg), potassium (600 mg), calcium (85 mg), niacin (1.1 mg), and phosphorus (50 mg) are all known to be present in bael fruit [29].

## 3. Phytochemicals

*Aegle marmelos* has been widely utilized in traditional medicinal systems. It has been reported to contain numerous photochemical compounds such as polyphenol/phenolic compounds, carotenoids, alkaloids, pectin, flavonoids, tannins, coumarins, and terpenoids (Table 2). Similarly, various bioactive compounds from these groups have been isolated and identified [41,42]. In the last few years, *A. marmelos* has attracted much attention due to its varied pharmacological activities such as anticancer, antioxidant, antidiabetic, cardioprotective, hepatoprotective, and antimicrobial activities. Therefore, it is important to determine/investigate the phytochemical profile of bael fruit to discover new sources for the development of new drugs and nutraceuticals. Various phytochemical studies are depicted in Table 2.

### 3.1. Polyphenol/Phenolic Compounds

Polyphenolic compounds are very important plant constituent as it is responsible for antioxidant activity. In a study, total phenolic content (TPC) in *A. marmelos* fruit was estimated using reversed-phase high performance liquid chromatography (RP–HPLC) method. From the findings of the study, it was estimated that value TPC is equal to 10.6 mg GAE/g [43]. Similarly, polyphenol content in bael fruit at various development and/or growth conditions was determined [45]. The authors determined that polyphenol content in bael fruit ranges from 5.21% to 5.99% whereas the amount of tannic acid varied from 2.81 to 4.84 g 100/g at different development stages. In addition, significant variation in marmelosin (415.75–737 μg/g) in bael powder was noticed at various maturity stages [45]. In a study conducted on *A. marmelos,* different phenolic compounds such as chlorogenic acid, ferulic acid, ellagic acid, gallic acid, quercetin, and protocatechuic acid in amounts of 136.8, 98.3, 248.5, 873.6, 56.9, and 47.9 µg/g, respectively were characterized through LC-MS and LC-MS/MS scans and HPLC method [46]. Comparable results were also obtained by Hazra et al. [12] in bael fruit under different conditions such as microwave dried sample (BM), sun-dried sample (BS), hot air-dried sample (BH), and freeze-dried sample (BF). From the findings, it was observed that TPC varies significantly due to drying with maximum in BM (25.14 GAE mg/g) and minimum in BH sample (16.23 GAE mg/g). In addition, TFC in BP varies from 1.16 g CE/100 g. Flavonoids and phenolic acids in *A. marmelos* fruit were also studied by Hazra et al. [12]. From different samples of bael fruit, a total of five phenolic acids, namely chlorogenic acid, gallic acid, p-coumaric acid, vanillic acid, and 2,3-dihydroxybenzoic acid, were detected in BH, BM, BS, and BF samples. The authors reported that gallic acid was maximum in BP (617.17 ± 2.58 mg/100 g), 2,3-dihydroxybenzoic acid in BH (35.94 mg/100 g), chlorogenic acid (CGA) in BM (56.31 mg/100 g), p-Coumaric acid (p-CA) in (361.42 mg/100 g) and vanillic acid (VA) was maximum in BS (102.40 mg/100 g). On the other hand, rutin (flavonoid) was found highest in BM (59.90 mg/100 g) and lowest in BP (32.25 mg/100 g) [12]. Other studies conducted in the last few years have also confirmed the presence of flavonoids and phenolic acids in bael fruit extract/pulp [42,51]. From the results, it was concluded that amount/content of different compounds varies with the method of drying. It was revealed that there are very limited studies related to the investigation and quantification of phytochemical compounds in bael fruit. Therefore, it is necessaryto investigate further for future benefit.

### 3.2. Coumarins

Bael fruit is considered a rich source of imperatorin, marmelosin, marmin, marmesin, alloimperatorin, marmelide, methyl ether, scoparone xanthotoxol, umbelliferone, scopolentin, and psoralen [52]. Marmenol has also been reported in bael fruit [53]. From the findings of the previous years, it has been reported that the bael fruit contains marmelosin, umbeliferone, imperation, scoporone, alloimperatorin, marmelide, marmesin, impertonin, umbelliferine, skimmianine, scopoletin, methyl ether, psoralen, marmin, xanthotoxol and armelide in considerable amounts. It is also determined that the coumarins such as umbelliferone marmelosin and skimmianine are recognized as medicinally important active principle compounds of bael fruit [54,55].

### 3.3. Carotenes

*A. marmelos* fruit have been reported to contain carotenoids, imparting yellow color to the fruit. Charoensiddhi and Anprung [44] have determined total carotenoids in the pulp of bael fruit that is equal to 32.98 μg/g dw. Similarly, Hazra et al. [12] have determined and identified different carotenoid in fruit of *A. marmelos*, viz., α-carotene (42.76–1698.22 μg/100 g), β-carotene (51.67–153.43 μg/100 g), γ-carotene (18.43–467.17 μg/100 g) and δ-carotene (43.74–45.03 μg/100 g). However, there are very few studies related to the determination of carotenoid content in bael fruit.

### 3.4. Other Phytochemicals

*A. marmelos* fruit have been investigated to determine and/or identify various phytochemicals present in its fruit. Compounds such as alkaloids (such as Aegelenine, Halfordinol, Aegeline, Ethyl cinnamate, Aegelinosides A, Ethyl cinnamamide, Aegelinosides B, Dictamine, and Fragrine), terpenoids (such as Caryophyllene, Valencene, Cineol, Terpinolene, cis-Limonene oxide, P-cymene, cis-Linalool oxide, Methyl perilate, Cubedol, Isosylvestrene, Elemol, Myrcene, Epi-cubebal, Humulene, Hexanylhexanoate, Linalool, and Limonene), tannins and polysaccharides (such as galactose, L-rahaminose, and arabinose) have been reported/determined from bael fruit [47,48,49,56,57]. However, there is lack of quantification data/information on these above-mentioned and other compounds. Therefore, it is necessary to conduct thorough investigation on the determination, identification and quantification of different phytochemicals present in bael fruit. Though from the above-mentioned studies, it has been confirmed that bael fruit is rich in active biological compounds that are responsible for various metabolic processes in the human body.

## 4. Pharmacological Activities of *Aegle marmelos*

### 4.1. Antidiarrheal Activity

Diarrhea is a common symptom of gastrointestinal infections and occurs mainly due to an imbalance of natural microflora of the gut by broad-spectrum antibiotics. The etiology of diarrhea has been widely studied in the past years, including pathogens involved in the same. An in vitro investigation was carried out to evaluate the antidiarrheal effect of *A. marmelos.* For this, the activity of ethanolic extract of dried *A. marmelos* fruit pulp was tested against pathogens, namely, *Shigella dysenteriae*, *S. boydii*, *S. flexneri*, and *S. sonnei*. From the findings, it was revealed that *S. dysenteriae* showed the minimum activity with a minimum inhibitory concentration (MIC) equal to 250 µg/mL and a minimum bactericidal concentration of 400 µg/mL. It was also concluded that ethanolic extract was found to be more effective at the lower end of the concentration tested (0.5–1.0 mg/mL) [58]. In another study, the antidiarrheal activity of *A. marmelos* (unripe fruit extract) was studied on castor oil-induced diarrhea in mice animal models at 400 mg/kg and 800 mg/kg BW. The authors reported that doses of *A. marmelos* ethanolic fruit extract significantly reduce (*p* < 0.05) a considerable number of wet feces produced due to treatment of mice with castor oil (Table 3). The inhibition frequency of defecation by fruit extract at 400 mg/kg and 800 mg/kg was evaluated to be 67.44% and 70.93%, respectively [59]. Similarly, methanolic extract of *A. marmelos* fruit was tested on castor oil-induced diarrhea in the SD rat animal model. The results revealed that methanolic extract showed a 100% inhibition rate, except in the first hour (78.13%), against diarrhea in an animal model [60]. However, in another independent study, it was investigated that the antidiarrheal activity of *A. marmelos* fruit is due to calcium channel blocking compounds and not tannic acid [61]. The authors reported that methanolic extract of *A. marmelos* ripe fruit inhibited diarrhea caused due to castor oil in mice animal models. The reduction (%) of wet feces and the total number of feces showed dose-dependent activity, whereas a dose of 400 mg/kg and 800 mg/kg showed substantial inhibition. Tannic acid (extracted from fruit *A. marmelos*) does not show an antidiarrheal effect but is more likely to act as vasorelaxant in mice, with a significant relaxant effect (EC_50_ = 0.1527 μM, 95% C.I., 0.005853–3.986) [61]. In another study, extract of *A. marmelos* was tested for its effect on colonization of *E. coli* E134, *E. coli* B170, and *S. flexneri*. The results showed that there is a decrease in colonization, perhaps due to its influence on the metabolism of HEp-2 cells and/or due to modification of cell receptors that limit bacterial adherence, as seen in the pre-incubation of HEp-2 with the extract. The extract exhibits greater inhibition of adherence of *S. flexneri* and *E. coli* E134 as compared to invasion of *E. coli* B170. Because pathogen adhesion to the lining of the gastrointestinal tract is the earliest stage of the illness process and inhibiting invasion/adherence could be a critical part of the antidiarrheal effect of the plant [47,62]. From the previous studies conducted on *A. marmelos* for determining antidiarrheal activity, it was estimated that it could be due to active phytochemical compounds such as alkaloids, saponins, tannins, and flavonoids present in fruit extract. Therefore, further investigations are required to confirm the exact mechanism of antidiarrheal activity shown by fruit extract of *A. marmelos*.

### 4.2. Antioxidant Activity

Antioxidants protect the body against the side effects of free radicals, which are responsible for number of health-related disorders such as heart disorders, high blood pressure, cancer, and diabetes. In a study, it was observed that fruit (pulp) extract of bael showed great antioxidant potential [50]. The authors reported that both alcoholic and aqueous extract of fruit produced more (44.36%) or less (40.12%) DPPH anion radical scavenging activity at dose of 100 μg/mL with IC_50_ value for both aqueous and alcoholic extract equal to 92.648 μg/mL and 106.15 μg/mL, respectively. While alcoholic and aqueous extract show reducing power (Fe^3+^ to Fe^2+^) equal to 28.7% and 50.33% at 100 μg/mL, with IC_50_ value of 283.06 µg/mL and 158.99 µg/mL, respectively. The ethanolic and aqueous fruit pulp extract exhibit substantial free radical scavenging activity, against NO (nitric oxide) with inhibition of 52.02% and 63.74% at 100 μg/mL and extract was also capable of scavenging H_2_O_2_ in a dose-dependent manner reaching from 73.77% (aqueous extract) to 69.0% (ethanolic extract) with IC_50_ equal to 56.53 μg/mL (aqueous extract) and 52.19 μg/mL (alcoholic extract) [50]. In another study, the methanolic extract of bael fruit was evaluated for its antioxidant activity via, DPPH and FRAP (ferric-reducing antioxidant power) assay. From the findings, it was determined that fruit extract show IC_50_ value of 52.06 µg/mL DW and 59.32 µmol/g DW for DPPH and FRAP assay, respectively. The authors of the above-mentioned study also compared the reducing capacity of fruit extract with leaves extract of *A. marmelos* and the results showed that fruit extract shows higher scavenging activity as compared to the leaves (*p* < 0.05) (IC_50_ = 46.5 µmol/g DW) [63]. While in a study conducted by Wijewardana et al. [64], scavenging activity (DPPH assay) of *A. marmelos* fruit powder ranged from 24.31% to 81.33% at concentration 200 to 1000 μg/mL of methanolic extract [64]. A study conducted on methanolic extract of unriped *A. marmelos* fruit shows that fruit extract is active against DPPH free radical scavenging, as evidenced by its IC_50_ equal to 62.59 μg/mL [39]. Similarly, chloroform and aqueous extract of dry and ripe *A. marmelos* fruit show significant free radical quenching activity (reducing ferric chloride), ranging from 88% to 65% at 5–0.15 μ/mL extract concentration [41]. Based on the findings of the studies, it was determined that the antioxidant potential of *A. marmelos* may be associated with the phytochemicals present in fruit, such as phenols, flavonoids and tannins. The antioxidant activity of *A. marmelos* supports that fruit may be used as antioxidant agent to treat cellular damage caused due to free radicals and it can be used as adjuvant with other drugs to increase effectiveness.

### 4.3. Antidiabetic Activity

Hyperglycaemia or high blood sugar level is typical complication of uncontrolled diabetes, leading to serious body damage, mainly affecting nerves and blood vessels [76,77]. Recently, fruit of *A. marmelos* has attracted much attention due to its uses in traditional medicinal system, but there are very limited studies related to its biological properties. In a study, fruit extract of *A. marmelos* was studied for its antidiabetic effect. The findings of the study reported that, fruit extract showed significant increase in (*p* > 0.001) in BWG (body weight gain) % (30.41–32.80) and FER (feed efficiency ratio) (0.087–0.096), while decrease in DFI (daily feed intake) (26.50–22.54 g/rat/day) when tested against alloxan diabetic rats [65]. Further, it was observed that, administration of *A. marmelos* fruit extract orally at dose of 125 mg/kg, 250 mg/kg and 500 mg/kg exhibit significant elevation in sugar (glucose) concentration (*p* > 0.001; 97.48–78.82 mg/dL) and reduction in insulin level (*p* > 0.01; 6.58–15.64 μIU/mL), when compared with untreated diabetic rats [65]. Interestingly to the food intake, administration of alloxan to normal rats, showed hyperphagia due to low arterio-venous gradient, as the cells cannot process glucose due to absence of insulin hormone, which may be responsible for hyperglycemia. Though, effect of bael fruit extract in preventing weight loss and improving food efficiency seems to be due to its ability to reduce hyperglycemia [65,78]. In another study, aqueous extract of bael fruit (AMFEt) was tested in female albino Streptozotocin (STZ)-induced diabetic Wistar rats and normal rats [66]. The study involves administration of STZ (45 mg/kg) intraperitoneally to induce diabetes in Wistar rats and AMFEt (250 mg/kg) two times daily for an interval of one month. From the findings, it was observed that there was major decrease in plasma insulin (*p* < 0.05) and significant increase in glucose level in blood (*p* < 0.05) in diabetic rats. While oral treatment of AMFEt decreases the blood glucose level (*p* < 0.05, 280.0–61.4 mgdL^–1^), increases plasma insulin level (17.9–21.6 µUmL^–1^) and improve body weight (178.6–194.0 g), food intake (51.2–54.8 g/day) and water intake (212.5–230.0 mL/day) in diabetic group and normal group, but no substantial change was observed in normal group (*p* < 0.05) [66]. From the results of the above-mentioned studies, it was revealed that *A. marmelos* fruit extract has significant effect on plasma insulin and blood glucose levels. Therefore, these studies shows that fruit of *A. marmelos* can be used as antidiabetic agent. However, there are very limited studies related to the investigation of antidiabetic effect of *A. marmelos*. Though, there is need to investigate further for the development of antidiabetic products or drugs.

### 4.4. Hepatoprotective Activity

The liver is an important organ involved in the detoxification and disposition of toxic substances. It is exposed to a wide range of chemotherapeutic, hepatotoxins, and xenobiotic agents, which leads to damage and significantly involves impairment of its function and metabolism [79,80]. In a literature survey, it was observed that the extract of bael fruit shows significant hepatoprotective activity. In a study carried out by Rajasekaran et al. [67], a step was taken forward to investigate the hepatoprotective effect of *A. marmelos* fruit. From the results, the authors confirmed that aqueous and ethanolic fruit extract showed moderate to significant protection activity. Ethanolic extract (500 mg/kg; *p* < 0.01) was observed to have moderate activity for serum glutamate pyruvate transaminase (SGPT; 64.5 U/mL), serum glutamate oxaloacetate transaminase (SGOT; 81.3 U/mL) and alkaline phosphatase (ALP; 8.1 KA units) in CCl_4_-induced liver damaged mice [67]. However, ethanolic extract of bael fruit holds the ability to restore normal functioning of the damaged liver caused due to CCl_4_ treatment; therefore, it could be used as a hepatoprotective agent. In a different study, *A. marmelos* fruit was tested for its effects in Wistar albino rats against cisplatin-induced hepatotoxicity [68]. The authors confirmed that upon administration of a diet containing (fruit) *A. marmelos* causes restoration of antioxidant status (*p* < 0.05) with reduction (*p* < 0.05) and increase in superoxide dismutase, catalase, lipid peroxidation, and glutathione and concentration in cisplatin-induced hepatotoxicity in tested animal models. In addition, administration of *A. marmelos* (2–4%) diets to model animals significantly reduces alanine aminotransferase (ALT), acid phosphatase (ACP), ALP, aspartate aminotransferase (AST), and bilirubin serum concentration levels [68]. However, it was concluded that the hepatoprotective effect of bael fruit could be due to its antioxidant potential, evident by increasing enzymatic and also reduction in serum levels as it was noticed that cisplatin treatment causes a reduction in enzymatic activity (antioxidant) and elevation of liver damage marker enzymes [68]. However, the *A. marmelos* diet shows protection against cisplatin-induced liver damage. Similarly, in a different study conducted by Sastry et al. [69], the hepatoprotective potential of aqueous *A. marmelos* fruit was investigated. From the findings, it was observed that due to paracetamol (2 g/kg) treatment on Wistar albino rats, the elevated levels of serum parameters were reduced (*p* < 0.001) significantly after administration/treatment with fruit extract at 100–400 mg/kg BW in a dose-dependent manner, i.e., ALP (123–168 IU/L), Bilirubin (BLN; 1.5–1.22 mg/dL), SGPT/ALT (43–54.33 IU/L), and SGOT/AST (176–218.3 IU/L) [69]. Therefore, the results of all the studies clearly indicated that the fruit of *A. marmelos* is effective in the treatment/prevention of hepato-cytotoxicity in model animals. Though, there is a need to investigate further in the same field, as there are very limited studies related to the hepatoprotective effect of *A. marmelos* fruit extract.

### 4.5. Radio Protective Activity

A study was conducted in which the hydroalcoholic fruit pulp extract of *A. marmelos* was examined for its radioprotective effects, which indicated that bael fruit is of medical and nutritional benefit [81,82]. Swiss albino mice animal model was administered a range of doses (5, 10, 20, 40, or 80 mg/kg) intraperitoneally (i.p.) for 5 successive days before being subjected to 10 Gy (exposure dosage) of gamma-radiation. Only 20 mg/kg groups showed a substantial increase in survival, with 50% (on 10 days) and 29% (on 30 days) survival after irradiation (*p* < 0.001). Dose-dependent studies were also carried out by administering either a placebo or 20 mg/kg bael fruit extract before exposure to irradiation (6–11 Gy). The LD_50/30_ for the group that was exposed to radiation alone was 8.2 Gy, while the LD _50/30_ for the group administered bael fruit extract before exposure to radiation was 8.8 Gy. As the DRF was found to be insignificant (1.1), no further research on the fruit extract was conducted [70]. In another study, the radioprotective effect of hydroalcoholic *A. marmelos* fruit extract (AME) was evaluated in mice exposed to varying amounts of radiation. The radioprotection optimal dose was found by giving AME i.p. 5 days (one per day) before being exposed to 10 Gy of radiation. Dosage of 20 mg/kg of AME (5 days prior to irradiation) was found to be most effective in radioprotection, as supported by the largest number of survivors after 30 days. Treatment of tested animals with AME before exposure to radiation reduced the effects of radiation sickness symptoms and mortality across all levels of radiation. On comparing the AME + irradiation group to the simultaneous sterile physiological saline (SPS) + irradiation group, the former group had a higher number of survivals. As shown by the higher number of survivors on days 10 and 30, AME pretreatment provides protection against bone marrow and gastrointestinal mortality. For 10 Gy (*p* < 0.001) and 9 (*p* < 0.05) irradiation, pretreatment with AME reduced 10-day mortality by 2- and 1.4-fold, respectively. With the 11 Gy dose of AME, a considerable % of the animals survived, but none of the animals in the contemporaneous control group survived past day 9 post-irradiation on day 10. For 9 and 8 Gy irradiation, pretreatment with AME reduced 30-day mortality by 2.6- and 1.2-fold, respectively. The treatment with AME prior to exposure to 10 Gy radiation resulted in a survival rate of 41.6%, while no survivors were reported in the concurrent control group. The LD_50/30_ was found to be 8.8 Gy for the AME+ irradiation group and 8.2 Gy for the SPS-treated group when the survival rate was plotted as log values v/s a linear irradiation dose scale [71].

### 4.6. Anticancer Activity

The anticancer potential of *A. marmelos* fruit extract was investigated in different studies. In a recent study, breast cancer was promoted in a rat animal (55–60 days old; 150 ±  10 g) model using 7,12-dimethylbenz(a)anthracene (DMBA; 20 mg/mL diluted in Olive oil). The ethanolic fruit pulp of *A. marmelos* was administered orally (200 mg/kg b.w./day) for the next five weeks after developing breast tumors (approximately 0.5 cm), and later, the amount of tumor was measured. Treatment with fruit extract of *A. marmelos* resulted in a reduction in breast tumor volume (*p* < 0.05), involving a significant drop (*p* < 0.0001) in serum biomarkers such as serum malondialdehyde (MDA), TNF-α, and glucose levels. After therapy with ethanolic fruit pulp extract, considerable (*p* < 0.0001) improvements in both liver and kidney serum biomarker values were detected. Taking everything into consideration, the ethanolic fruit pulp extract of *A. marmelos* displays anti-proliferative activity by slowing the progression of breast cancer in a tested animal model (rat). Hepato-renal protection is also a benefit of the plant extract. Therefore, it could be used as a new and safe anticancer therapy against breast cancer cell lines [72]. Natural phytochemical substances are being used in cancer chemoprevention as a new way to prevent, postpone, or cure cancer. The MTT assay is a colorimetric method for determining cell metabolism, while MTT in vitro cell proliferation assay was used extensively for evaluating the preliminary cytoprotective efficacy of synthetic derivatives, natural compounds, and extracts obtained from natural products. This assay indicates whole-cell cytotoxicity; however, additional assays are required to determine the precise molecular target. Kinase enzymes play a significant role in a number of biological processes, and inhibitors of kinase enzymes have been reported to exhibit cytoprotective effects against various human cancer cell lines. The water-soluble MTT (3-(4,5-dimethylthiazol-2-yl) 2,5-diphenyltetrazolium bromide) is converted to insoluble formazan in the MTT experiment, followed by involving solubilization of formazan. The concentration is determined by optical density measurements at 570 nm. At a concentration of 100 g/mL of aqueous fruit pulp extract of *A. marmelos*, the maximal MCF7 cell death was 66.51%, and the IC_50_ was 47.92 µg/mL. The anticancer activity was measured using the MTT test method, which revealed an IC_50_ value of 47.92 µg/mL [73]. Chemotherapy seems to be the gold standard in cancer treatment for over 60 years and is still effective in the treatment of metastatic malignancies. However, the majority of therapeutically utilized chemotherapeutic drugs have intrinsic histological toxicity, reducing the remedial benefit [71]. The methanolic extract of the fruit has been demonstrated to have a cytotoxic impact (in vitro) on the SKBR3 cell line (human breast cancer cells) in preclinical studies. The IC_50_ was found to be 144.00 ± 1.21 μg/mL, indicating the pulp as an anticancer agent [74]. Chemoprevention is a strategy for cancer prevention that is based on a protective approach to cancer control [16]. Recent investigations in Swiss albino mice have revealed that a methanolic fruit pulp extract of *A. marmelos* has strong preventive properties against DMBA-induced cutaneous papilloma genesis. In the preinitiation phase (7 days before and after DMBA application), administration of the bael extract orally (50 mg/kg B.W.) resulted in a 70% reduction in tumor incidence, while the post-initiation phase (start of croton oil treatment to the end of the experiment) resulted in a 50% decrease in tumor prevalence. Bael fruit also decreased the overall number of tumors, frequency of occurrence per animal, and tumor production, implying that it could be used as a chemo-preventive drug [75].

## 5. Conclusions and Future Perspectives

Bael is being cultivated and consumed worldwide due to its highly nutritious value, delightful taste, and valuable biological activities. The review showed the brilliant composition of bioactive compounds in bael fruits, such as carbohydrates, proteins, various vitamins, fatty acids, and minerals, along with many phytochemicals including terpenoids, flavonoids, saponins, tannins, phenolic acids, and glycosides. The bael fruit has also proved to possess antidiarrheal, antioxidant, antidiabetic, hepatoprotective, radioprotective, and anti-cancerous properties in various experiments conducted in vivo and in vitro on human cell lines and animal models. The review highlighted the high potential of bael fruits in accordance with their nutritional value and useful bioactivities. The bael fruit can be used widely as powder, wine, preserve, jam, and juice, which can be very effective for patients with diabetes, oral-gastric ulcers, and cancer. Various clinical experiments have been undertaken to guarantee that the bael fruit is safe to consume, but knowledge of its physiology and bioactivities in humans is limited. Therefore, it is critical to conduct more research into the molecular mechanisms of bael fruit bioactivities to explore the applications of its compounds in food and pharmaceutical products. Considering all the concerns related to the safety of humans, clinical trials of bael fruit-based products will increase their value as food and pharma products by a huge margin, which might prove its potential worth in forthcoming days.

## Figures and Tables

**Table 1:** Nutritional composition of bael (*Aegle marmelos* (L.) Correa) fruit according to various studies.

Source/Region	Type	Compound	Yield/Concentration	Reference
Bael fruit pulp (Bangladesh)	Polysaccharide (%)	Carbohydrate	36.80–41.70	[21]
		Total sugar	3.08–6.94	
Bael fruit (North India)	Polysaccharide (%)	Fructose	1.01–1.55	[22]
		Glucose	1.15–1.88	
		Sucrose	2.45–12.01	
Bael fruit pericarp (Ludhiana, Punjab)	Polysaccharide (%)	Total sugars	1.83	[6]
		Non-reducing sugars	0.91	
		Reducing sugars	0.92	
Bael fruit pulp (Ludhiana, Punjab)	Polysaccharide (%)	Total sugars	14.35	
		Non-reducing sugars	9.93	
		Reducing sugars	4.42	
Bael fruit pulp (Karachi, Pakistan)	Polysaccharide (%)	Carbohydrate	34.35	[5]
Bael fruit pulp (Patiala, Punjab)	Polysaccharide (g/100 g)	Total soluble sugars	7.6	[23]
		Reducing sugars	6.2	
		Non-reducing sugars	1.4	
		Starch	3.6	
Bael fruit (Rajasthan, India)	Polysaccharide (%)	Carbohydrate	31.8	[24]
Bael fruit pulp (Bangladesh)	Protein (%)	-	7.52–8.81	[21]
Bael fruit pericarp (Ludhiana, Punjab)	Protein (%)	-	1.31	[6]
Bael fruit pulp (Ludhiana, Punjab)	Protein (%)	-	3.64	
Bael fruit pulp (Rajasthan, India)	Protein (%)	-	4.35	[25]
Bael fruit pulp (Karachi, Pakistan)	Protein (%)	-	1.87	[5]
Bael fruit Faizabad (U.P.) Nadia, West Bengal	Protein (%)	-	3.6	[1]
	Protein (%)	-	8.8	
Bael fruit pulp (Patiala, Punjab)	Protein (g/100 g)	-	4.7	[23]
Bael fruit	Protein (%)	-	1.8	[26]
Bael fruit (Rajasthan, India)	Protein (%)	-	1.8	[24]
Bael fruit pulp (Karachi)	Fat (%)	-	45.6	[5]
Bael edible portion	Fat (%)	-	0.6	[27]
Bael fruit pulp	Fat (%)	-	3.7	[28]
Bael (unripe fruit)	Minerals (mg)	P	52	[1,7]
		K	610	
		Ca	80	
Bael fruit pulp	Minerals (mg/100 g)	Cu	0.21	[1]
		K	610	
		Ca	80	
		P	52	
		Fe	0.60	
Bael fruit (Bellary district)	Minerals (mg)	Ca	85	[29]
		P	50	
		K	600	
	Vitamins (mg)	Vitamin A	55	
		Vitamin C	8–60	
		Riboflavin	1.19	
		Niacin	1.1	
Bael fruit	Vitamins (mg)	Vitamin A	55	[30]
		Vitamin C	8	
Bael fruit	Vitamins (mg/100 g)	Vitamin A	55	[30,31]
		Vitamin B_1_	0.13	
		Vitamin B_2_	1200	
		Vitamin C	8	
		Riboflavin	1190–1200	
		Thiamine	0.13	
		Ascorbic acid	8	
Bael fruit pulp (Karachi)	Vitamins (mg%)	Vitamin B_1_	0.16	[5]
		Vitamin B_2_	0.18	
		Vitamin B_3_	0.87	
		Vitamin C	73.2	

P—phosphorus; K—potassium; Ca—calcium; Fe—iron; Cu—copper.

**Table 2:** Phytochemical profile of *Aegle marmelos* (fruit).

Variety/Region	Type	Compound	Concentration/Yield	Reference
*A. marmelos*	TPC (mg GAE/g)	-	10.6–87.34	[43,44]
*A. marmelos* (Lucknow)	Polyphenol	Tannic acid (g 100/g)	2.81–4.84	[45]
		Marmelosin (μg/g)	415.75–737.00	
*A. marmelos*	Polyphenol (µg/g)	Chlorogenic acid	136.8	[46]
		Ferulic acid	98.3	
		Ellagic acid	248.5	
		Gallic acid	873.6	
		Quercetin	56.9	
		Protocatechuic acid	47.9	
*A. marmelos* (Kolkata, West Bengal, India)	TPC (GAE mg/g)	-	16.23–25.14	[12]
	TFC (g CE/100 g)	-	9.74–18.17	
	Phenolic acid (mg/100 g)	Gallic acid	580.27–617.17	
		2,3-dihydroxy benzoic acid	10.35–35.94	
		Chlorogenic acid	0.38–56.31	
		p-Coumaric acid	233.54–361.42	
		Vanillic acid	52.80–102.40	
	Flavonoid (mg/100 g)	Rutin	32.25–59.90	
	Carotenes (μg/100 g)	α-carotene (alpha C)	42.76–1698.22	
		β-carotene (beta C)	51.67–153.43	
		γ-carotene (gamma C)	18.43–467.17	
		δ-carotene (delta C)	43.74–45.03	
*A. marmelos* (Phichit, Thailand)	Total flavonoids (mg CE/g dw)	-	15.20 ± 0.51	[44]
	Total carotenoids (μg/g dw)	-	32.98 ± 0.51	[44]
*A. marmelos*	Alkaloids	Aegelenine, Halfordinol, Aegeline, Ethyl cinnamate, Aegelinosides A, Ethyl cinnamamide, Aegelinosides B, Dictamine, Fragrine	-	[7,47,48,49]
	Terpenoids	Caryophyllene, Valencene, Cineol, Terpinolene, cis-Limonene oxide, P-cymene, cis-Linalool oxide, Methyl perilate, Cubedol, Isosylvestrene, Elemol, Myrcene, Epi-cubebal, Humulene, Hexanylhexanoate, Linalool, Limonene	-	[49]
	Coumarins	Alloimperatorin, Zanthotoxol, Imperatorin, Xanthotoxol, Isoimperatorin, Umbelliferone, Marmelide, Scopoletin, Marmelosin, Scopolentin, Marmesin, Psoralen-a, Scoparone, Marmin, Methyl ether, Psoralen	-	
	Tannins	Skimminianine, 4,7,8-trimethoxyfuro-quinoline,	-	[6]
	Amino acids	Methionine, Phenyl alanine, Leucine, Tyrosine, Isoleucine, Alanine, Aspartic acid, and Arginine	-	[50]

CE = catechin equivalent; GAE = gallic acid equivalent.

**Table 3:** Pharmacological activities of *A. marmelos* fruit.

Variety/Region	Type of Study/Activity	Type of Extract	Organism or Cell Line/Assay	Dosage	Key Findings	Reference
*A. marmelos* (LallubhaiVrajalal Gandhi, Ahmedabad, India)	Antidiarrheal (in vitro)	Ethanolic extract of dried fruit pulp	*Shigella boydii*, *S. dysenteriae*, *S. sonnei* and *S. flexner*	0.5–4 mg/mL	MIC:MBC *Shigella sonnei*—250:500, Shigella flexner—400:500, Shigella boydii—500:500, *Shigella dysenteriae*—250:400. Maximum inhibition was observed for *Shigella sonnei* while minimum value of inhibition was observed for *Shigella dysenteriae*	[58]
*A. marmelos*	Antidiarrheal (in vivo)	Ethanolic extract of unripe fruit	Animal model: mice	400–800 mg/kg	Extract of unripe fruits of Aegle marmelos show inhibition of 67.44% and 70.93% at dosages of 400 mg/kg and 800 mg/kg, respectively	[59]
*A. marmelos*	Antidiarrheal (in vivo)	Methanolic extract of unripe fruit	Animal model: SD rat	15–1600 mg/kg orally	Significant inhibition against diarrhea induced by administration of castor oil in rats. No wet feaces were observed after 1 h.	[60]
*A. marmelos* (Tiruchirappalli, Tamilnadu, India)	Antioxidant	Aqueous and alcoholic (ethanol) extract of fruit pulp	-	100 µg/mL	DPPH assay—44.36–40.12% (IC_50_ = 92.648–106.15 μg/mL); ferric-reducing assay—28.7–50.33% (IC_50_ = 158.99–283.06 µg/mL); NO scavenging = 52.02–63.74%; H_2_O_2_ scavenging = 69.0–73.77% (IC_50_ = 52.19–56.53 μg/ mL)	[50]
*A. marmelos*	Antioxidant	Methanolic extract of fruit	-	-	DPPH assay—IC_50_ = 52.06 µg/mL DW; FRAP assay—IC_50_ = 59.32 µmol/g DW	[63]
*A. marmelos*	Antioxidant	Methanolic extract of fruit	-	200–1000 μg/mL	DPPH assay: 24.31–81.33%	[64]
*A. marmelos* (Delhi, India)	Antioxidant	Methanolic extract of unripe fruit	-	-	IC_50_ = 62.59 μg/mL (DPPH assay)	[39]
*A. marmelos*	Antioxidant	Chloroform and aqueous extract of dry and ripe fruit	-	5–0.15μ/mL	Scavenging activity = 88–65%	[41]
*A. marmelos*	Antidiabetic (in vivo)	Ethanolic extract of fruit	Animal model: Alloxan (120 mg/kg) induced diabetic rats (180–195 g)	Alloxan: 120 mg/kg; Ethanolic extract: 125–500 mg/kg/day	Glucose—97.48–78.82 (mg/dL); Insulin—6.58–15.64 (μIU/mL) Reduction in glucose level while increase in insulin level in serum with increase in dosage of A. marmelos fruit extract	[65]
*A. marmelos* (Vijayawada, Andhra Pradesh, India)	Antidiabetic (in vivo)	Aqueous extract of fruit	Female albino Streptozotocin (STZ) induced diabetic Wistar rats	AMFEt: 250 mg/kg; STZ: 45 mg/kg	Decrease in blood glucose level (*p* < 0.05, 280.0–61.4 mgdL^–1^) and increase in plasma insulin level (17.9–21.6 µUmL^–1^)	[66]
*A. marmelos* (Vellore, Tamil Nadu, India)	Hepatoprotective (in vivo)	Ethanolic extract of fruit pulp	CCl_4_-induced liver damage mice	Ethanolic extract—500 mg/kg	Reduction in SGOT: 81.3 U/mL; SGPT: 64.5 U/mL and ALP: 8.1 KA units levels in CCl_4_-treated animals	[67]
*A. marmelos* (Bilaspur, Chhattisgarh, India)	Hepatoprotective (in vivo)	Fruit pulp	Cisplatin-induced liver damage Wistar albino rat (91–129 g)	Cisplatin (6 mg/kg); Fruit extract concentration—2–4%	AST: 69.84–110.19 U/L ALT: 89.26–133.25 U/L ALP: 129.81–161.73 U/L ACP: 131.61–182.54 U/L (*p* < 0.05). Increasing activity of antioxidant enzymes, and significant change in physiological parameters	[68]
*A. marmelos* (Vijayawada, Andhra Pradesh, India)	Hepatoprotective (in vivo)	Aqueous fruit extract	Paracetamol-induced liver damage in Wistar albino rats	Paracetamol: 2 g/kg; Aqueous fruit extract 100–400 mg/kg	Reduction in level (*p* < 0.001) of ALP (123–168 IU/L), Bilirubin (BLN; 1.5–1.22 mg/dL), SGPT/ALT (43–54.33 IU/L) and SGOT/AST (176–218.3 IU/L)	[69]
*A. marmelos*	Radioprotective (in vivo)	Hydroalcoholic fruit extract	Age-matched Swiss albino mice	Dosage: 5–80 mg/kg	LD_50/30_ value recorded for the group administered with bael extract before exposure to radiation was 8.8 Gy. Mice administered with dosage of 20 mg/kg bael extract showed increase in survival with 50% and 29% survival after 10 and 30 days, respectively	[70]
*A. marmelos*	Radioprotective (in vivo)	Hydroalcoholic fruit extract	Mice	Dosage: 20 mg/kg	LD_50/30_ was recorded to be 8.8 Gy for mice administered with bael fruit extract. For 10 Gy (*p* < 0.001) and 9 Gy (*p* < 0.05) irradiation, pretreatment with AME reduced 10-day mortality by 2- and 1.4-fold, respectively	[71]
*A. marmelos*	Anti-cancerous (in vivo)	Ethanolic fruit pulp extract	Female Charles Foster rats (~150 g)	Dosage: 200 mg/kg BW/day	Decrease in breast tumor volume (*p* < 0.05), as well as a significant decrease (*p* < 0.0001) in serum biomarkers- serum malondialdehyde (MDA), TNF-α), and glucose levels	[72]
*A. marmelos*	Anti-cancerous (in vitro)	Aqueous fruit pulp extract	MCF7 cell line	Dosage: 100 g/mL	Maximal MCF7 cell death was at a rate of 66.514.65%, with IC_50_ value of 47.92 µg/mL	[73]
*A. marmelos*	Anti-cancerous (in vitro)	Methanolic extract of the fruit	Human breast cancer cells (SKBR3)	-	IC_50_ of 144.00 ± 1.21 μg/mL when bael fruit extract was tested against SKBR3	[74]
*A. marmelos*	Anti-cancerous (in vivo)	Methanolic extract of the bael fruit pulp	Swiss albino mice	Dosage: 50 mg/kg BW	Administration of bael fruit extract (orally) results in a 70% inhibition of tumor, with 50% reduction in tumor incidence during post-initiation phase	[75]

MBC = minimum bactericidal concentration; MIC = minimum inhibitory concentration.; DPPH = 2,2-diphenyl-1-picryl-hydrazyl-hydrate).

## Data Availability

Not applicable.

## References

[1] Singh A., Sharma H.K., Kaushal P., Upadhyay A. (2014). Bael (*Aegle marmelos* Correa) products processing: A review. Afr. J. Food Sci..

[2] Jhajhria A., Kumar K. (2016). Tremendous pharmacological values of *Aegle marmelos*. Int. J. Pharm. Sci. Rev. Res..

[3] Neeraj V.B., Johar V. (2017). Bael (*Aegle marmelos*) extraordinary species of India: A review. Int. J. Curr. Microbiol. Appl. Sci..

[4] Shaikh R.U., Ahmed A.A. (2021). Medicinal Flora of Poona College.

[5] Lakht-e-Zehra A., Dar N.G., Saleem N., Soomro U.A., Afzal W., Naqvi B., Jamil K. (2015). Nutritional exploration of leaves, seed and fruit of bael (*Aegle marmelos* L.) grown in Karachi region. Pak. J. Biochem. Mol. Biol..

[6] Kaur A., Kalia M. (2017). Physico chemical analysis of bael (*Aegle Marmelos*) fruit pulp, seed and pericarp. Chem. Sci. Rev. Lett..

[7] Sarkar T., Salauddin M., Chakraborty R. (2020). In-depth pharmacological and nutritional properties of bael (*Aegle marmelos*): A critical review. J. Agric. Food Res..

[8] Singh A.K., Singh S., Saroj P.L., Singh G.P. (2021). Improvement and production technology of bael (*Aegle marmelos*) in India—A review. Curr. Hortic..

[9] Maity P., Hansda D., Bandyopadhyay U., Mishra D.K. (2009). Biological activities of crude extracts and chemical constituents of bael, *Aegle marmelos* (L.) Correa. Ind. J. Exp. Bio..

[10] Asghar N., Mushtaq Z., Arshad M.U., Imran M., Ahmad R.S., Hussain S.M. (2018). Phytochemical composition, antilipidemic and antihypercholestrolemic perspectives of Bael leaf extracts. Lipids Health Dis..

[11] Tagad V.B., Sahoo A.K., Annapure U.S. (2018). Phytochemical study and GC-MS analysis of bael (*Aegle marmelos*) fruit pulp. Res. J. Life Sci. Bioinform. Pharm. Chem. Sci..

[12] Hazra S.K., Sarkar T., Salauddin M., Sheikh H.I., Pati S., Chakraborty R. (2020). Characterization of phytochemicals, minerals and in vitro medicinal activities of bael (*Aegle marmelos* L.) pulp and differently dried edible leathers. Heliyon.

[13] Punia S., Kumar M. (2021). Litchi (*Litchi chinenis*) seed: Nutritional profile, bioactivities, and its industrial applications. Trends Food Sci. Technol..

[14] Dutta A., Lal N., Naaz M., Ghosh A., Verma R. (2014). Ethnological and Ethno-medicinal importance of *Aegle marmelos* (L.) Corr (Bael) among indigenous people of India. Am. J. Ethnomed..

[15] Roy S.K., Saran S., Kitinoja L. (2011). Bael (*Aegle marmelos* (L.) Corr. Serr.). Postharvest Biology and Technology of Tropical and Subtropical Fruits.

[16] Baliga M.S., Bhat H.P., Joseph N., Fazal F. (2011). Phytochemistry and medicinal uses of the bael fruit (*Aegle marmelos* Correa): A concise review. Food Res. Int..

[17] Bel-Rhlid R., Berger R.G., Blank I. (2018). Bio-mediated generation of food flavors–Towards sustainable flavor production inspired by nature. Trends Food Sci. Technol..

[18] Sonawane A., Pathak S., Pradhan R.C. (2020). Bioactive compounds in bael fruit pulp waste: Ultrasound-assisted extraction, characterization, modeling, and optimization approaches. Biointerface Res. Appl. Chem..

[19] Lovegrove A., Edwards C.H., De Noni I., Patel H., El S.N., Grassby T., Shewry P.R. (2017). Role of polysaccharides in food, digestion, and health. Crit. Rev. Food Sci. Nutr..

[20] Lee C.H., Chen K.T., Lin J.A., Chen Y.T., Chen Y.A., Wu J.T., Hsieh C.W. (2019). Recent advances in processing technology to reduce 5-hydroxymethylfurfural in foods. Trends Food Sci. Technol..

[21] Sarkar A., Rashid M., Musarrat M., Billah M. (2021). Phytochemicals and Nutritional Constituent Evaluation of Bael (*Aegle marmelos*) Fruit Pulp at Different Development Stage. Asian Food Sci. J..

[22] Yadav N., Singh P., Mehrotra R. (2011). Determination of some ethnomedicinally important constituents of *Aegle marmelos* fruit during different stages of ripening. Chin. J. Nat. Med..

[23] Singh U., Kocher A., Boora R. (2012). Proximate composition, available carbohydrates, dietary fibres and anti-Nutritional factors in Bael (*Aegle marmelos* L.) leaf, pulp and seed powder. Int. J. Sci. Res. Publ..

[24] Rathore M. (2009). Nutrient content of important fruit trees from arid zone of Rajasthan. J. Hortic. For..

[25] Sharma K., Chauhan E.S. (2016). Nutritional and phytochemical evaluation of fruit pulp powder of *Aegle marmelos* (Bael). J. Chem. Pharm. Res..

[26] Kumar K.S., Umadevi M., Bhowmik D., Singh D.M., Dutta A.S. (2012). Recent trends in medicinal uses and health benefits of Indian traditional herbs *Aegle marmelos*. Pharma Innov..

[27] Apou A.C., Farzana T., Suzauddula M., Hossain M. (2019). The Physicochemical Characteristics and Quality Evaluation of Bael Fruit (Limoniaacidissima L.) Pulp Powder for the Product of Functional Food. Daffodil Int. Univ. J. Allied Health Sci..

[28] Gopalan C., Rama Sastri B.V., Balasubramanian S.C. (1971). Nutritive Value of Indian Foods.

[29] Ullikashi K.Y., Kammar M.R., Lokapure S.R. (2017). Development of value added products from bael fruit (*Aegle marmelos*). Int. J. Curr. Micro Appli. Sci..

[30] Murthy H.N., Bhat M.A., Dalawai D. (2020). Bioactive compounds of bael (*Aegle marmelos* (L.) correa). Bioactive Compounds in Underutilized Fruits and Nuts.

[31] Venthodika A., Chhikara N., Mann S., Garg M.K., Sofi S.A., Panghal A. (2021). Bioactive compounds of *Aegle marmelos* L., medicinal values and its food applications: A critical review. Phyther. Res..

[32] Wu G. (2016). Dietary protein intake and human health. Food Funct..

[33] Rejman K., Górska-Warsewicz H., Kaczorowska J., Laskowski W. (2021). Nutritional Significance of Fruit and Fruit Products in the Average Polish Diet. Nutrients.

[34] Villa-Rodriguez J.A., Yahia E.M., González-León A., Ifie I., Robles-Zepeda R.E., Domínguez-Avila J.A., González-Aguilar G.A. (2020). Ripening of ‘Hass’ avocado mesocarp alters its phytochemical profile and the in vitro cytotoxic activity of its methanolic extracts. S. Afr. J. Bot..

[35] Rios J.L. (2016). Essential Oils: What They Are and How the Terms Are Used and Defined. Essential Oils in Food Preservation, Flavor and Safety.

[36] Alabi K.P., Zhu Z., Sun D.W. (2020). Transport phenomena and their effect on microstructure of frozen fruits and vegetables. Trends Food Sci. Technol..

[37] Bazaid S.A., El-Amoudi M.S., Ali E.F., Abdel-Hameed E.S. (2013). Volatile oil studies of some aromatic plants in Taif region. J. Med. Plants Stud..

[38] Mahato H., Kumar B. (2022). Medicinal Uses with Immense Economic Potential and Nutritional Properties of *Aegle marmelos*: A Concise Review. Biocomposites.

[39] Gupta D., John P.P., Kumar P., Jain J. (2018). Evaluation of antioxidant activity of unripe *Aegle marmelos* Corr. Fruits. J. Appl. Pharm. Sci. Res..

[40] Bhardwaj R.L., Nandal U. (2015). Nutritional and therapeutic potential of bael (*Aegle marmelos* Corr.) fruit juice: A review. Nutr. Food Sci..

[41] Rahman S., Parvin R. (2014). Therapeutic potential of *Aegle marmelos* (L.)—An overview. Asian Pac. J. Trop. Dis..

[42] Manandhar B., Paudel K.R., Sharma B., Karki R. (2018). Phytochemical profile and pharmacological activity of *Aegle marmelos* Linn. J. Integr. Med..

[43] Wali A., Gupta M., Mallick S.A., Gupta S., Jaglan S. (2016). Antioxidant Potential and Phenolic Contents of Leaf, Bark And Fruit Of" *Aegle marmelos*". J. Trop. For. Sci..

[44] Charoensiddhi S., Anprung P. (2008). Bioactive compounds and volatile compounds of Thai bael fruit (*Aegle marmelos* (L.) Correa) as a valuable source for functional food ingredients. Int. Food Res. J..

[45] Gurjar P.S., Bhattacherjee A.K., Singh A., Dikshit A., Singh V.K. (2019). Characterization of nutraceuticals in bael powder prepared from fruits harvested at different developmental stages. Indian J. Tradit. Knowl..

[46] Prakash D., Upadhyay G., Pushpangadan P., Gupta C. (2011). Antioxidant and free radical scavenging activities of some fruits. J. Complement. Integr. Med..

[47] Bhar K., Mondal S., Suresh P. (2019). An eye-catching review of *Aegle marmelos* L. (Golden Apple). Pharmacogn. J..

[48] Poonkodi K., Vimaladevi K., Suganthi M., Gayathri N. (2019). Essential oil composition and biological activities of *Aegle marmelos* (L.) correa grown in Western Ghats Region-South India. J. Essent. Oil Bear. Plants.

[49] Pathirana C.K., Madhujith T., Eeswara J. (2020). Bael (*Aegle Marmelos* L. Corrêa), a Medicinal Tree with Immense Economic Potentials. Adv. Agric..

[50] Rajan S., Gokila M., Jency P., Brindha P., Sujatha R.K. (2011). Antioxidant and phytochemical properties of *Aegle marmelos* fruit pulp. Int. J. Curr. Pharm. Res..

[51] Panda S.K., Sahu U.C., Behera S.K., Ray R.C. (2014). Bio-processing of bael [*Aegle marmelos* L.] fruits into wine with antioxidants. Food Biosci..

[52] Farooq S. (2005). 555 medicinal plants. Field and Laboratory Manual (Identification with Its Phytochemical and In Vitro Studies Data).

[53] Kokate C.K., Purohit A.P., Gokhale S.B. (2002). Text Book of Pharmacognosy.

[54] Sharma P.C., Bhatia V., Bansal N., Sharma A. (2007). A review on Bael Tree. Nat. Prod. Radiance.

[55] Bramhachari P.V., Reddy Y., Kotresha D., Varaprasad B. (2010). Phytochemical examination, antioxidant and radical scavenging activity of *Aegle marmelos* (L.) Correa extracts. J. Pharm. Res..

[56] Rishabha M., Ajay K., Anupama S., GT K. (2012). Pharmacological screening, Ayurvedic values and commercial utility of *Aegle marmelos*. Int. J. Drug Dev. Res..

[57] Patel P.K., Sahu J., Sahu L., Prajapati N.K., Dubey B.K. (2012). Aegle marmelos: A review on its medicinal properties. Int. J. Pharm. Phytopharm. Res..

[58] Joshi Y., Chaudhary R.K., Teotia U.V.S. (2013). Formulation and evaluation of diclofenac sodium sustained release matrix tablets using *Aegle marmelos* gum. Int. J. Curr. Trends Pharm. Res..

[59] Mehesare S.S., Waghmare S.P., Thorat M.G., Hajare S.W., Itankar P.R., Ali S.S. (2019). Evaluation of the antidiarrhoeal activity of extract of unripe fruit of *Aegle marmelos*. J. Pharmacogn. Phytochem..

[60] Ghorai S., Sarma K., Choudhury P.R., Das G., Singh D., Kalita G., Choudhury J., Arya R.S. (2018). Anti-Diarrhoeal Activity and Toxicity Trial of Methanolic Fruit-Pulp Extract of *Aegle Marmelos* (L.) Correa in Sprague-Dawle Rats. Int. J. Livest. Res..

[61] Taqvi S.I.H., Rahman A., Versiani M.A., Imran H., Khatoon A., Sohail T. (2018). Studies to determine antidiarrhoeal and spasmolytic activities of extract of *Aegle marmelos*. L fruit. Bangladesh. J. Med. Sci..

[62] Brijesh S., Daswani P., Tetali P., Antia N., Birdi T. (2009). Studies on the antidiarrhoeal activity of *Aegle marmelos* unripe fruit: Validating its traditional usage. BMC Complement. Altern. Med..

[63] Andleeb R., Ijaz M.U., Rafique A., Ashraf A., Bano N., Zafar N., Ahmedah H.T. (2021). Biological Activities of Methanolic Extract of *Aegle marmelos* against HN Protein of Newcastle Disease Virus. Agronomy.

[64] Wijewardana R.M.N.A., Nawarathne S.B., Wickramasinghe I., Gunawardane C.R., Wasala W.M.C.B., Thilakarathne B.M.K.S. (2016). Retention of physicochemical and antioxidant properties of dehydrated bael (*Aegle marmelos*) and palmyra (*Borassus*
*flabellifer*) fruit powders. Procedia Food Sci..

[65] Abdallah I.Z., Salem I., El-Salam A., Nayrouz A.S. (2017). Evaluation of antidiabetic and antioxidant activity of *Aegle marmelos* L. Correa fruit extract in diabetic rats. Egypt. J. Hosp. Med..

[66] Kamalakkanan N., Rajadurai M., Prince P., Stanely M. (2003). Effect of *Aegle marmelos* Fruits on Normal and Streptozotocin-Diabetic Wistar Rats. J. Med. Food.

[67] Rajasekaran C., Kalaivani T., Ramya S., Jayakumararaj R. (2009). Studies on hepatoprotective activity of ethanolic extracts of fruit pulp of *Aegle marmelos* (L.) Corr. J. Pharm. Res..

[68] Chandel S.S., Shirsat M., Sahu R.K., Nayak S.S. (2018). Modulatory effect of dietary inclusion of *Aegle marmelos* fruits against cisplatin-induced hepatotoxicity in Wistar rats. Ann. Hepatol..

[69] Sastry A.V.S., Girija S.V., Naga S.G., Srinivas K. (2011). Phytochemical investigations and hepatoprotective effects of Aqueous fruit extract of *Aegle marmelos* Corr. Int. J. Chem. Sci..

[70] Baliga M.S., Bhat H.P., Pereira M.M., Mathias N., Venkatesh P. (2010). Radioprotective effects of *Aegle marmelos* (L.) Correa (Bael): A concise review. J. Altern. Complement. Med..

[71] Jagetia G.C., Venkatesh P., Baliga M.S. (2004). Fruit extract of *Aegle marmelos* protects mice against radiation-induced lethality. Integr. Cancer Ther..

[72] Akhouri V., Kumari M., Kumar A. (2020). Therapeutic effect of *Aegle marmelos* fruit extract against DMBA induced breast cancer in rats. Sci. Rep..

[73] Vardhini S.P., Sivaraj C., Arumugam P., Ranjan H., Kumaran T., Baskar M. (2018). Antioxidant, anticancer, antibacterial activities and GC-MS analysis of aqueous extract of pulps of *Aegle marmelos* (L.) Correa. J. Phytopharmacol..

[74] Moongkarndi P., Kosem N., Luanratana O., Jongsomboonkusol S., Pongpan N. (2004). Antiproliferative activity of Thai medicinal plant extracts on human breast adenocarcinoma cell line. Fitoterapia.

[75] Agrawal A., Verma P., Goyal P.K. (2010). Chemomodulatory effects of *Aegle marmelos* against DMBA-induced skin tumorigenesis in Swiss albino mice. Asian Pac. J. Cancer Prev..

[76] Giri B., Dey S., Das T., Sarkar M., Banerjee J., Dash S.K. (2018). Chronic hyperglycemia mediated physiological alteration and metabolic distortion leads to organ dysfunction, infection, cancer progression and other pathophysiological consequences: An update on glucose toxicity. Biomed. Pharmacother..

[77] Daryabor G., Atashzar M.R., Kabelitz D., Meri S., Kalantar K. (2020). The effects of type 2 diabetes mellitus on organ metabolism and the immune system. Front. Immunol..

[78] Guyton A., Hall J.E., Hall J.E. (2011). Dietary Balances; Regulation of Feeding; Obesity and Starvation; Vitamins and Minerals. Guyton and Hall Textbook of Medical Physiology.

[79] Mohi-Ud-Din R., Mir R.H., Sawhney G., Dar M.A., Bhat Z.A. (2019). Possible pathways of hepatotoxicity caused by chemical agents. Curr. Drug Metab..

[80] Jetter A., Kullak-Ublick G.A. (2020). Drugs and hepatic transporters: A review. Pharmacol. Res..

[81] Sharma G.N., Dubey S.K., Sharma P., Sati N. (2011). Medicinal values of bael (*Aegle marmelos*) (L.) Corr.: A review. Int. J. Curr. Pharm. Rev. Res..

[82] Benni J.M., Jayanthi M.K., Suresha R.N. (2011). Evaluation of the anti-inflammatory activity of *Aegle marmelos* (Bilwa) root. Indian J. Pharmacol..

